# Antioxidant, Antiproliferative, and Antiangiogenesis Effects of Polyphenol-Rich Seaweed (*Sargassum muticum*)

**DOI:** 10.1155/2013/604787

**Published:** 2013-09-04

**Authors:** Farideh Namvar, Rosfarizan Mohamad, Javad Baharara, Saeedeh Zafar-Balanejad, Fahimeh Fargahi, Heshu Sulaiman Rahman

**Affiliations:** ^1^Institute of Tropical Forestry and Forest Products (INTROP), Universiti Putra Malaysia (UPM), 43400 Serdang, Selangor, Malaysia; ^2^Department of Medicine & Applied Biology Research Center, Mashhad Branch, Islamic Azad University, Iran; ^3^Faculty of Biotechnology and Biomolecular Sciences, Universiti Putra Malaysia (UPM), 43400 Serdang, Selangor, Malaysia; ^4^Department of Biology, Applied Biology Research Center, Mashhad Branch, Islamic Azad University, Iran; ^5^Department of Microbiology and Pathology, Faculty of Veterinary Medicine, Universiti Putra Malaysia (UPM), 43400 Serdang, Selangor, Malaysia

## Abstract

In the present study, we evaluated the effect of brown seaweeds *Sargassum muticum* methanolic extract (SMME), against MCF-7 and MDA-MB-231 breast cancer cell lines proliferation. This algae extract was also evaluated for reducing activity and total polyphenol content. The MTT assay results indicated that the extracts were cytotoxic against breast cancer cell lines in a dose-dependent manner, with IC_50_ of 22 **μ**g/ml for MCF-7 and 55 **μ**g/ml for MDA-MB-231 cell lines. The percentages of apoptotic MCF-7-treated cells increased from 13% to 67% by increasing the concentration of the SMME. The antiproliferative efficacy of this algal extract was positively correlated with the total polyphenol contents, suggesting a causal link related to extract content of phenolic acids. Cell cycle analysis showed a significant increase in the accumulation of SMME-treated cells at sub-G1 phase, indicating the induction of apoptosis by SMME. Further apoptosis induction was confirmed by Hoechst 33342 and AO/PI staining. Also SMME implanted in vivo into fertilized chicken eggs induced dose-related antiangiogenic activity in the chorioallantoic membrane (CAM). Our results imply a new insight on the novel function of *Sargassum muticum* polyphenol-rich seaweed in cancer research by induction of apoptosis, antioxidant, and antiangiogenesis effects.

## 1. Introduction

Seaweed is one of the most extensively used functional foods and medicinal herbs with a long history in Asian countries. It refers to a wide variety of different species with different medicinal activities which is divided into two groups, namely, microalgae and macroalgae or seaweed. The medical use of seaweeds goes back at least 5,000 years to ancient China [1]. Seaweeds are known as functional food because of their richness in lipids, minerals and certain vitamins, and also several bioactive substances like polysaccharides, proteins, and polyphenols, with potential medicinal uses against cancer, oxidative stress [2], inflammation [2], allergy [3], diabetes [4], thrombosis [5], obesity [6], lipidemia [7], hypertensive [8], and other degenerative diseases. Since certain seaweeds have long been used in the treatment of cancer, many kinds of crude or partially purified polysaccharides from various brown and red algae were tested for their property and showed antitumor activity against experimental tumour [9, 10]. The epidemiological data are supported by animal model studies showing protective effects of dietary algae against skin [11], intestinal [12], and mammary cancer [13, 14]. The anticancer potency of mechanism through which algae exert their effects is complex because of their noteworthy structural diversity, which entails multiple interactions [15, 16]. An algal antioxidant-mediated mechanism [17, 18] enhances the host's defense by increasing natural killer cell activity [19], activating of nonspecific immune system [9], inhibiting the cell growth in the G1 phase, inducing terminal differentiation [7], inhibiting the complex process of angiogenesis [20], down regulating the endogenous oestrogen biosynthesis [14], and inducting of apoptosis [14] which were hypothesized as a contributing factors in the inhibition of carcinogenesis by algae.

Angiogenesis means formation of a vascular network from the previous vessels, which occurs in different pathologic conditions such as tumor growth and metastasis, and it has also an important role in some physiologic processes like the organ growth, wound healing, and reproduction. All solid tumours are dependent on the angiogenesis in order for the tumour to grow larger and metastasize [21]. Endothelial cells in the tumor bed due to their high proliferation rate tend to be more susceptible to cytotoxic agents. Additionally, endothelial cells, on the contrary to cancerous cells, are genetically stable as they do not undergo mutations and thus are more sensitive to apoptosis effects of the cytotoxic agents [22]. Therefore, these features of endothelial cells make them a convincing target for antiangiogenesis treatment [22]. Consequently, cytotoxic agents pose as candidates as for antiangiogenic agents on top of their potent activity in causing death of cancerous cells. Alternatively, extensive studies have been conducted to assess the role of oxidative stress and hence the use of antioxidants in the prevention of cancer. In the initial stage of cancer, oxidative stress plays a major role in damaging essential components of cells [23]. Furthermore, several antiangiogenic drugs are currently being under clinical trials. But most of these angiogenic inhibitors are in essential concerns with side effects in animals and humans. Considering these perspectives, there have been extensive studies on natural product compounds and extracts that showed potent antiangiogenic activity, in conjunction with having good antioxidant activities to overcome the adverse effect of synthetic compounds on human health.

Marine macroalgae are the potential renewable resource on the marine environment, which have been reported to afford several beneficial effects. Chemical and nutritional composition of seaweeds varies with individuals, species, habitats, and maturity and depends on geographical origin or area of cultivation, seasonal, environmental, and physiological variations, and water temperature [24]. On the other hand, the effect of algal components on angiogenesis remained unknown or insufficient. The recent increasing demand for seaweed products, as anticancer drugs, and numerous mechanisms of modulation of carcinogenesis by seaweed are which have not been established definitively justify this investigation on the antioxidant, antiproliferative, and antiangiogenesis effects of polyphenol-rich seaweed (*Sargassum muticum*) obtained from Persian Gulf waters. To the best of our knowledge, this is the first report on the biofunctional properties of *Sargassum muticum *from Persian Gulf waters.

## 2. Materials and Methods

### 2.1. Raw Material

 Specimens of the seaweed from the coastal areas of Persian Gulf waters were washed and stored at −20°C. Ground, freeze-dried seaweed samples were methanol-extracted [25], filtered, and rotary-evaporated at 40°C to give a viscous mass (stored at −20°C).

### 2.2. Chemical

The 3-(4,5-dimethylthiazolyl-2)-2,5-diphenyltetrazolium bromide (MTT) was purchased from Sigma-Aldrich Canada (Oakville, ON). RPMI 1640 growth medium, L-glutamine, sodium bicarbonate, nonessential amino acids, sodium pyruvate, fetal bovine serum (FBS), and phosphate-buffered saline (PBS) were obtained from Invitrogen Corporation (Burlington, ON), and methanol was from Merck (Darmstadt, Germany). All reagents were of analytical grade.

### 2.3. Evaluation of Antioxidant and Total Phenolic Content

 The Ferric reducing antioxidant power assay (FRAP) procedure which is described by Benzie and Strain was followed [26]. 

Total phenolic was determined calorimetrically using Folin-Ciocalteu reagent as described by Velioglu et al. [27] with slight modifications. HPLC was used for compound identification of SMME which is described before [14].

### 2.4. Cell Line and Cell Culture

A normal cell line Vero cell (ATCC CCl-81, African green monkey kidney cell line) was used to test the cytotoxic effect of SMME. Vero cell was cultured in the growth medium (GM) that contained the Eagle's Minimum Essential Medium (MEM, GIBCO, USA), supplemented with 10% fetal bovine serum (FBS), 0.1% gentamicin, 20 mM HEPES, and 2 mM of glutamine, harvested and plated in flat bottom 96-well plates at the density of 1.5 × 10³ cell/mL in GM, and incubated at 37°C in a humidified incubator containing 5% CO_2_ for 24 h. After removing old medium, the cells were treated with varying concentrations (0, 200 *μ*g/mL) of test samples solution in MEM containing 1% FBS (MM) and incubated at 37°C for 3 days. Cells overlaid with only MM were used as control. The morphological alterations of the cells in their shape and level of adhesion were inspected daily under inverted microscope (Nikon CMM 214, Japan). The maximal nontoxic concentration (MNCC), defined as the maximal concentration of the extract that did not exert toxic effect detected by microscopic monitoring, was recorded at the end of treatment.

Human cancer cell lines, purchased from the American Type Culture Collection (ATCC, Rockville, MD, USA), were cultured in RPMI 1640 medium (GIBCO, USA) and supplemented with FBS and gentamicin as earlier. The antiproliferative activities were determined by the MTT assay. In brief, cells were plated in 96-well plates (1 × 10³ cells per well), incubated for 24 h at 37°C, were treated with various concentrations of seaweed extract, and incubated for 24, 48, and 72 h. MTT solution was added and the optical density was read with an ELISA reader. 

### 2.5. Morphological Assessment of Apoptosis

In this experiment, morphological alterations induced by SMME were examined using phase contrast, fluorescence, and electron microscopy.

In each T plate, treated and untreated cells were washed with PBS, fixed, dehydrated, then mounted onto stubs using standard procedure before coating with gold in a sputter coater for 1.5 min, and viewed immediately using SEM or stored in a silica gel desiccators.

The adhered cells were detached, solidified, fixed, dehydrated, and then infiltrated with acetone: resin mixture, using standard procedures and were embedded for polymerization. Thick sectioning (1 *μ*m) was performed using glass knife on the ultramicrotome. The sections were stained and examined under light microscope for ultrathin sectioning, and specific areas were selected, picked up, stained, and washed and finally were stained with lead acetate and washed with double distilled water before viewing under the transmission electron microscope.

MCF-7 cells (plated at a density of 5 × 10^5^ cells in a 35 mm dish) were exposed to GCME and then harvested with the propidium iodide (PI) DNA stain according to the kit protocol, or stained with acridine orange (AO) and PI for observation of chromatin condensation. The percentage of viable, necrotic, and apoptotic population for each time point was determined from more than 500 cells. The populations of each cell cycle phase were counted by flow cytometry (FACScan flow cytometer with Cell Quest software).

Treated and untreated cells were fixed, washed, and incubated for 15 min at 37°C with Hoechst 33342 dye (5 *μ*g/mL in PBS). Cells are visualized using an Olympus BHZ, RFCA microscope (Japan) equipped with a fluorescent light source with an excitation wavelength of 330 nm and a barrier filter of 420 nm. A minimum of 200 cells is counted at the same preparation but five different areas. Experiments are repeated at least three different times and classified in the following criteria: live cells: normal nuclei and blue/green pale chromatin with organized structure and apoptotic cells: early apoptotic cells can be identified by the presence of chromatin condensation within the nucleus and intact nuclear boundaries, bright blue chromatin that is highly condensed, marginated; late apoptotic cells exhibit nuclear fragmentation into smaller nuclear bodies within an intact cytoplasmic membrane.

### 2.6. Caspase Assay

The protease activity of caspases-3, -8, and -9 in MCF-7 cells was performed using colorimetric assay kit (GenScript kit, code: L00289, Piscataway, NJ 08854, USA) that is based on spectrophotometric detection of the caspase enzymes after cleavage from the labeled substrate. About 2 × 10^6^ MCF-7 cells were treated with SMME at IC_50_ and incubated for 24, 48, and 72 h, while untreated cells incubated for 24, 48, and 72 h acted as control. Then, the cells were centrifuged for 5 min at 2000 rpm to remove the media. Followed by the cells which were washed two times with PBS and centrifuged at 2000 rpm for 5 min. The cell pellets were lysed by the addition of 50 *μ*L cold prepared lysis buffer containing 0.5 *μ*L DTT and 0.25 *μ*L PMSF, mixed well, and incubated on ice for exactly 1 h. During this time, tubes were vortexed with vibration 3-4 times for 10 sec each time. The resulting cell lysate was centrifuged for 1 min at 10000 rpm at 4°C, and the supernatant was collected. Protein concentrations in each tube were quantified using Bradford method. Then, 50 *μ*L 2x reaction buffer containing 0.5 *μ*L DTT and 0.25 *μ*L PMSF were added to 50 *μ*L supernatant containing 200 *μ*g protein in each tube, to which 5 *μ*L caspase substrate was added, transferred to 96-well plate, wrapped, and incubated at 37°C for 4 h away from light. At the end of the incubation period, the samples were read at 405 nm in a microplate reader (Universal Microplate Reader) (Biotech, Inc., USA). Data was presented as optical density (OD) (405 nm; mean SD) and histogram was plotted. 

### 2.7. CAM Assay

For Antiangiogenic effect, 40 fertilized eggs were distributed randomly into 4 groups including the control group, the sham exposed (treated with DMSO solvent), and the experimental groups of 1 and 2 (treated with SMME 50 and 100 *μ*g/mL). The fertilized eggs were placed at the incubation which rotated automatically at the temperature of 38°C and relative humidity of 55–65%. In the second day of incubation, at the completely sterilized condition, which was created by Laminar Hood (Telstar, Spain), a part of the egg shells was removed and a window was made at one side of the eggs, which was closed by lamel and sterilized paraffin. Afterwards, the eggs were returned to the incubator, and manual rotation was performed twice a day for natural development of the chicks. Since the chorioallantoic membrane of the chicks starts its creation from the 5th day of incubation and in the 8th day occupies more than half of the eggs, and also because in this day the heart is divided and the vein and the artery separation happens, the treatment of the vessel network at the 8th day can be noted. Therefore, in the 8th day of incubation, the window of the shells was removed as completely sterilized condition, and a gelatin sponge containing the albumen and agar solution in normal saline (with equal ratio) with 200 *μ*L of streptomycin penicillin, wchich was prepared freshly at sterilized condition, were placed on the chorioallantoic membrane of the chicks at 4 × 4 mm. In the samples treated with SMME, 10 mL of extract was added to the gelatin sponge, and then the places of the windows were again covered and the eggs were returned to the incubator. On the 12th day of incubation, all of the samples (control, sham exposed, experimentally treated with concentration of 50 and 100 *μ*L/mL), the pictures were taken from the region of the gelatin sponge location by means of the research photostereomicroscopy (Zeiss, Germany). The parameters under investigation are the number and the length of the blood vessels that were measured around the gelatin sponge in all of the samples. Since the chorioallantoic membrane has a wide dick-like anatomic structure with thickness of 400 *μ*m, all the countable existing blood vessels in the squares around the sponges were considered. 

### 2.8. Statistical Analysis

All data were expressed as means ± SEM. One-way analysis of variance (ANOVA; SPSS 16.0 for Windows; SPSS Inc., Chicago, IL, USA) was used to test for differences between different treatment concentrations. Where differences did exist, the source of the differences at a *P* < 0.05 significance level was identified by the Student-Newman-Keuls multiple range tests. 

## 3. Results and Discussion

The results of antioxidant activity of SMME by using ferric reducing antioxidant power (FRAP) assay shows 75.32 ± 11.36 mmol, Fe II per 100 g dried plant. Total phenolic contents of SMME were 78.95 ± 4.33 mg gallic acid equivalents per 100 g dried plant. The genus *Sargassum* is tropical and subtropical brown seaweed comprising 150 species. The phenolic compounds were one of the most effective antioxidant in brown algae and its content 20–30% algae dry weight [28, 29]. Based on absorption profile, the retention time and spiking tests on the SMME, catechin, phlorotannins, and quercetin were among the phenolic compounds in SMME. Phlorotannins were one of the three major peaks that were identified. Phlorotannins are the main phenolic compounds detected in brown which is divided into three parts, soluble phlorotannin from algal matrix or cytoplasmic phlorotannin, cell-wall-bound phlorotannin that attached to the membrane, or cell wall and exuded phlorotannin that exude into the surrounding seawater. The antioxidant activity of the membrane-bound extract was higher than cytoplasmic extract [28]. Phlorotannins are 10–100 times more potent and more stable antioxidants than are other polyphenols; also, the half-life of the phlorotannins in the body is up to 12 h, compared to 30–180 min for terrestrial polyphenols [14].

Oxidative stress forced by reactive oxygen species (ROS) plays a critical role in the pathophysiology associated with chronic disease such as neoplasia [30]. The ROS-induced development of cancer involves malignant transformation due to DNA mutations and altered gene expression through epigenetic mechanisms. Extensive attention has been focused on identifying naturally occurring antioxidative phenolic phytochemicals that are able to decrease ROS levels. Phenolic compounds mostly found in plants are reported to have numerous biological effects including antioxidant, antiapoptosis, antiaging and anticarcinogenic properties and are therefore considered for their important dietary roles as antioxidants and chemoprotective agents. Recently, much attention has been paid to marine organisms for the screening of biologically active compounds. Among these marine organisms, seaweeds are considered to be very attractive sources, due to their enormous biodiversity and safety and as they have long been used in traditional Asian foods. Although several reports have suggested that crude seaweed extracts have antiproliferative activity in cancer cell lines, most studies focused on their antioxidant activity. The present study is the first to report the antioxidant, antiproliferative, and antiangiogenesis activities of extracts from the Persian Gulf brown (*Sargassum muticum*) seaweed.

The results of the cytotoxicity tested by observing the cellular morphological change showed that SMME had no toxicity to normal cell Vero cell line, but dose- and time-dependently inhibited the proliferation of the two breast cancer cell lines. The IC_50_ values calculated for SMME on MCF-7 cells were 20 ± 0.1 and for MDA-MB-231 were 55 ± 0.2 *μ*g/mL after treatment for 24 h, respectively. These results confirmed that the extracts of seaweeds selectively inhibited the growth of particular cell types or tumour types. In the report of Harada and Noro, 47 species of alga exhibited strong cytotoxic activity against L1210 cells. They also showed similar cytotoxicity with low cytotoxicity to normal cells. Such selective activity was also conspicuous in other seaweeds reported in their experiment. All these results together with this study suggested that the active substances interact with special cancer-associated receptors or cancer cell special molecule, thus triggering some mechanisms that cause cancer cell death [31].

Previous studies reported that hot water extracts of several brown algae were effective against some mouse cancer cells, and the active principle was found to be polysaccharide fraction. Among 306 species of seaweeds involved in the screening experiment of Harada and Noro, a remarkably selective cytotoxic activity was found in the MeOH extract from a red alga, *Amphiroa sonata*. In this study, it is reported that the chemical properties of the active substance present in the extract were heat stable and soluble in relatively higher polar organic solvents and water. The molecular weight was less than 3000, which shows that it is different from the substances such as polysaccharides reported from other studies [32]. 

Induction of apoptosis is a useful approach in cancer therapies. In apoptotic cells, several cellular and molecular biological features, such as cell shrinkage, DNA fragmentations, and activation of the caspase cascade, are exhibited [33].

**Figure 1:**
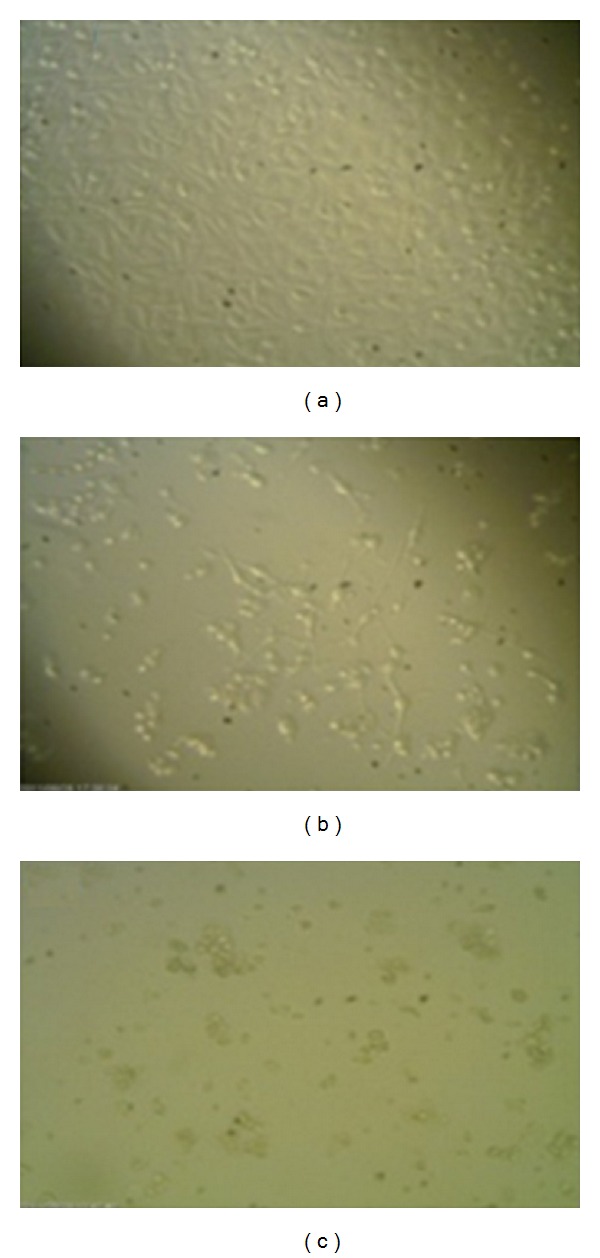
Morphological changes of MCF-7 cells under inverted microscope. (a) Control, (b) MCF-7 cells treated with 20 *μ*g/mL SMME, and (c) MCF-7 cells treated with 100 *μ*g/mL SMME (200x).

**Figure 2:**
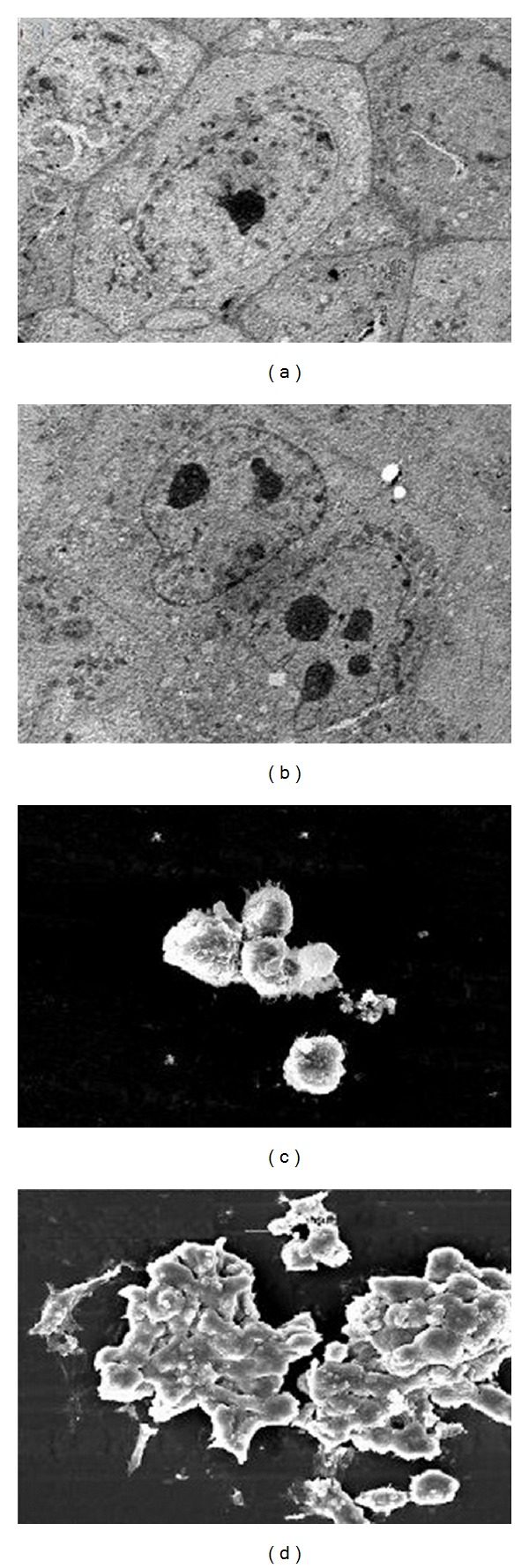
Morphological changes of MCF-7 cells under TEM and SEM microscope. (a) Control cells under TEM, (b) MCF-7-treated cells under TEM, (c) control cells under SEM, and (d) MCF-7-treated cells under SEM.

**Figure 3:**
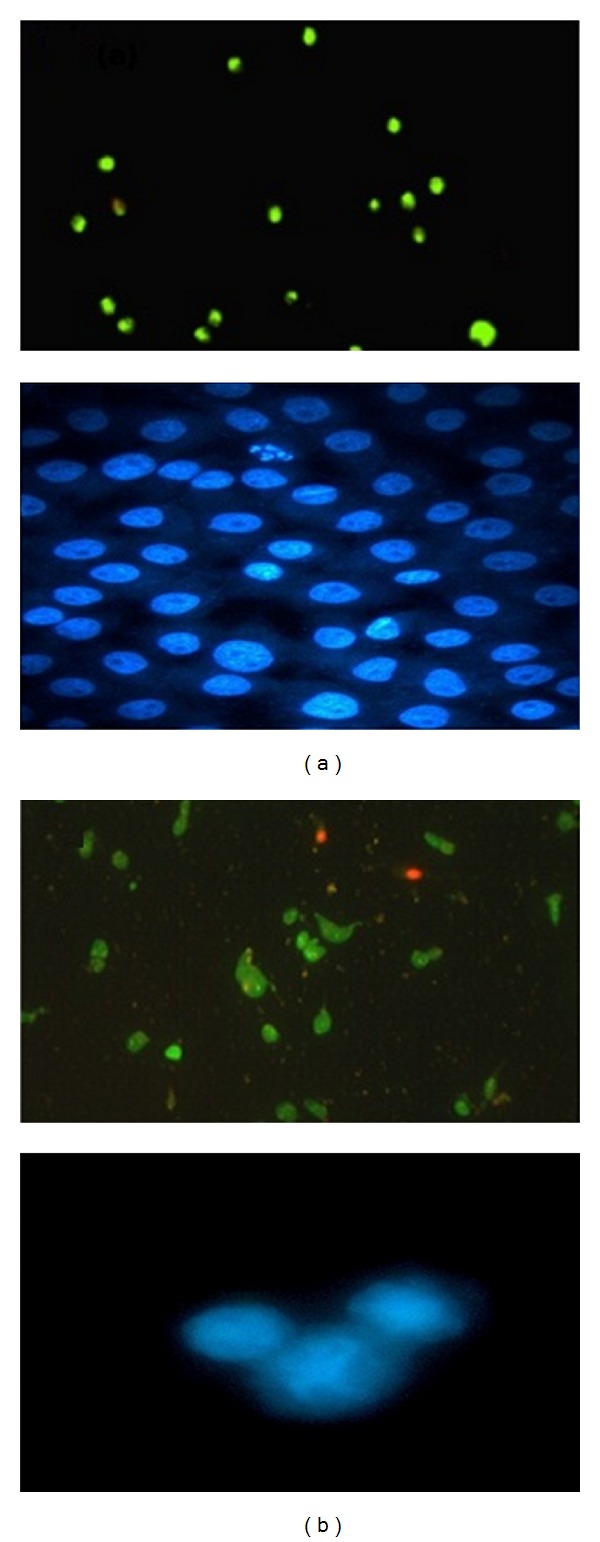
Morphological changes of MCF-7 cells stained with AO/PI or Hoechst 33342 under fluorescence microscope. (a) Control and (b) cell blebbing in treated MCF-7 cell with SMME.

The series of photographs in Figures 1–3 illustrates some of the possible morphologies seen with Invert, SEM, TEM, AO/PI staining, and Hoechst 33342 nuclear staining and fluorescence microscopy. 

In observation under EM, typical apoptotic characteristics were found, including cell membrane blebbing, microvillus disappearance or reduction, and separated apoptotic bodies (Figure 2). In addition, treated MCF-7 cells were observed under TEM, and shrinkage of cells, and condensation of chromosomes were found (Figure 2). 

Cytotoxicity of marine algal extracts was evaluated by growth inhibition (Figure 3). When the growth inhibited cells were stained with AO/PI and Hoechst 33342 apoptotic cell death was observed in time- and dose-dependent manner in all cultures (Figure 3). These results suggested that SMME caused irreversible cell damages in cultured cells.

**Figure 4:**
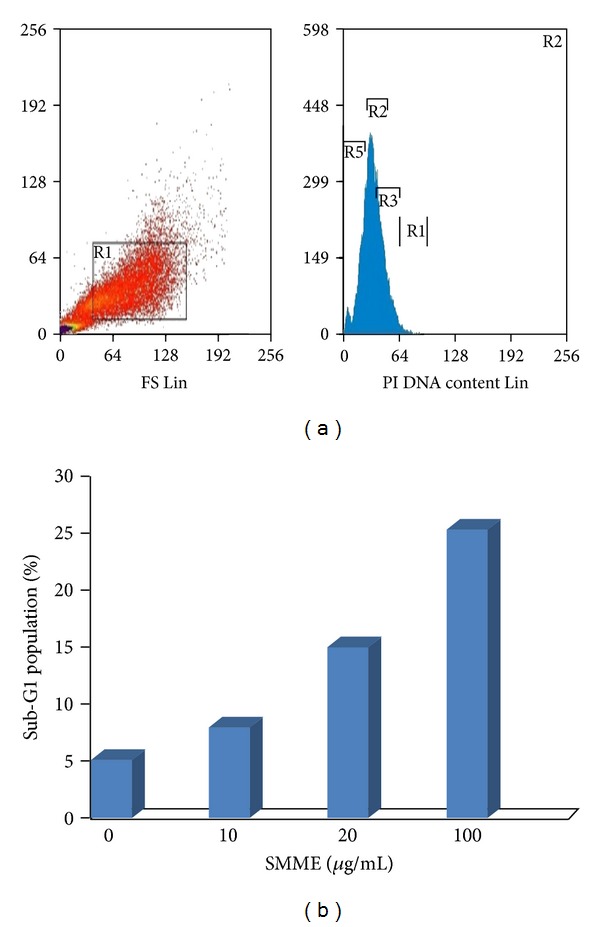
Cell cycle analysis of MCF-7 cells treated with SMME by flow cytometry. MCF-7 cells were incubated with various concentrations of SMME for 48 h. The cells were then stained with PI and analyzed by flow cytometry.

Intact membranes exclude PI but allow the uptake of AO, which binds to double-stranded DNA and fluoresces green under 488 nm excitation. Untreated cells show a diffuse green fluorescence, while in apoptotic cells, condensed chromatin material resulted in clumps of intense green fluorescent spots within the cell. The characteristic condensation patterns observed were the crescent shape at the nuclear periphery and the more numerous round clumps. Purely necrotic cells stain red with PI with no green fluorescence evident. At specific time intervals (24, 48, and 72 hours), the proportion of normal, apoptotic, and necrotic cells was scored as a percentage of the total cell population counted. In MCF-7 cells treated with SMME, apoptotic cells increased from 0.8% before treatment to 49% after 24 hours, 61% after 48 hours, and 74% after 72 hours. Percentages of apoptotic cells increased from 13% to 67% by increasing concentration of seaweeds extract from 10 to 100 *μ*g/mL (Figure 4). This increase did not exist in control cells. 

In cells treated with apoptosis-inducing agents, a subpopulation of cells appears before the G1 peak and is referred to as the sub-G1 peak, which is believed to be the result of endonuclease activation and subsequent leakage of DNA from the cells. Necrotic cells do not show the immediate reduction in DNA content, so a distinction can be made. As shown in Figure 4, the sub-G1 population, which indicated apoptotic cells, increased in a dose-dependent manner from 5.15% at 0 *μ*g/mL (control) to 8.10% at 20 *μ*g/mL, 15.1% at 50 *μ*g/mL, and 25.4% at 100 *μ*g/mL, after exposure to SMME for 48 h. Although the G1 population decreased along with an increase of sub-G1, the other portion of nonapoptotic cells did not show a significant change. Regulation of the cancer cell cycle is one strategy in the development of anticancer drugs. Kwon et al. have also reported that the ethanolic extracts of *Corallina pilulifera* showed cytotoxic activity against human cervical adenocarcinoma cell line, HeLa. Their results also showed that the ethanolic extracts of *Corallina pilulifera* can induce apoptosis in HeLa cells without cell cycle arrest [34]. Moreover, it is also reported that the *L. japonica* water extract induced apoptosis in several human breast cancer cell lines, potentially related to inhibition of SOD activity by iodine, or the effects of other compounds such as polyphenols [13]. A role for algal polyphenols as anticarcinogens and antiproliferative agents is further supported by antitumour promotion activity against ornithine decarboxylase induction by tumour promoter 12-O-tetradecanoylphorbol-13-acetate (TPA) in BALB/c 3T3 fibroblasts with 75–87% inhibition by *Laminariales sp. *and 92% inhibition by *P. tenera* methanol extracts [35]. A remarkable selective cytotoxic activity was found in methanolic extract from a red alga, *Gracilaria cortica* compared to *Ulva fasciata* and *Sargassum ilicifolium* in this study. 

The other research group demonstrated that seaweed induced apoptosis in human breast cancer cells with greater potency than that of fluorouracil, a chemotherapeutic agent used to treat breast cancer. This finding led the authors to speculate that seaweed may be applicable for prevention of breast cancer [13].

**Figure 5:**
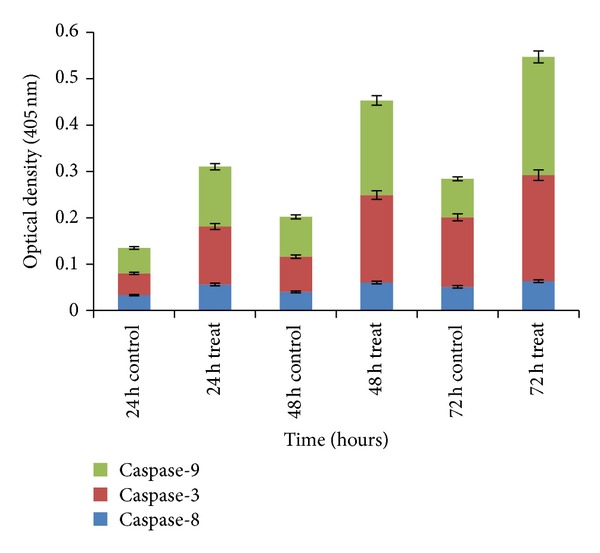
Activity of caspases-3, -8, and -9. The total cell lysates from MCF-7 cells treated with SMME for 24, 48 h, and 72 h were analyzed using caspase colorimetric protease assay kits.

The induction of apoptosis essentially needs the activation of caspases. Caspases, a group of intracellular proteases, are responsible for planning the cell into apoptotic bodies during apoptosis. Caspases are present as inactive proenzymes that are activated by proteolytic cleavage. Caspases-8, -9, and -3 are situated at key junctions in apoptosis pathways. As shown in Figure 5, SMME increased the activity of caspases-8, -9, and -3 in a time-dependent manner. According to the studies concerning caspase activities, several distinct pathways exist resulting in the induction of apoptosis by seaweed. In this work, we noted that SMME activate caspase-3 in a time-response fashion (Figure 5). These findings affirmed the results reported previously which have shown that FCSPs (“fucoidan”) from *F. vesiculosus* induce apoptosis in human lymphoma HS-Sultan cell lines and in HT-29 and HCT116 human colon cancer cells in vitro, and moreover that the exposure of these cells to the *F. vesiculosus* FCSPs appears to activate caspase-3. The apoptosis induced by seaweed via the activation of caspase-3 was reported previously to be mediated through a mitochondrial pathway [36]. Caspase-8 initiates disassembly in response to extracellular apoptosis-inducing ligands and is activated in a complex associated with the cytoplasmic death domain of many cell surface receptors for the ligands. Caspase-9 activates disassembly in response to agents or insults that trigger the release of cytochrome c from mitochondria and is activated when complexed with apoptotic protease activating factor-1 (APAF-1) and extramitochondrial cytochrome c. Caspase-3 appears to amplify caspase 8 and caspase 9 initiation signals into full-fledged commitment to disassembly. Caspase 8 and caspase 9 activate caspase 3 by proteolytic cleavage, and caspase-3 then cleaves vital cellular proteins or other caspases.

**Figure 6:**
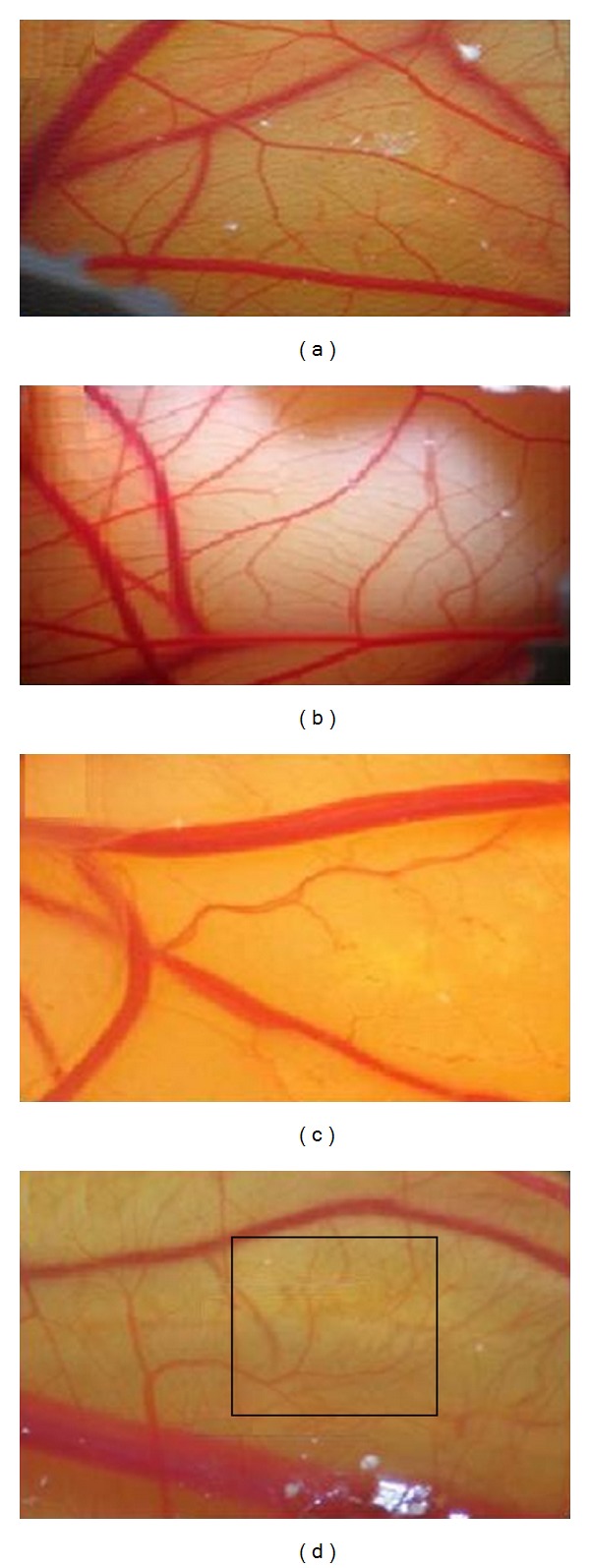
Photographs of the inhibitory effect of SMME on vascularization of the 12-day CAM. (a) and (b) chorioallantoic membrane related to the control group, (c) and (d) Chorioallantoic membrane related to the treated group. Black quadrant shows one of the measured areas.

**Figure 7:**
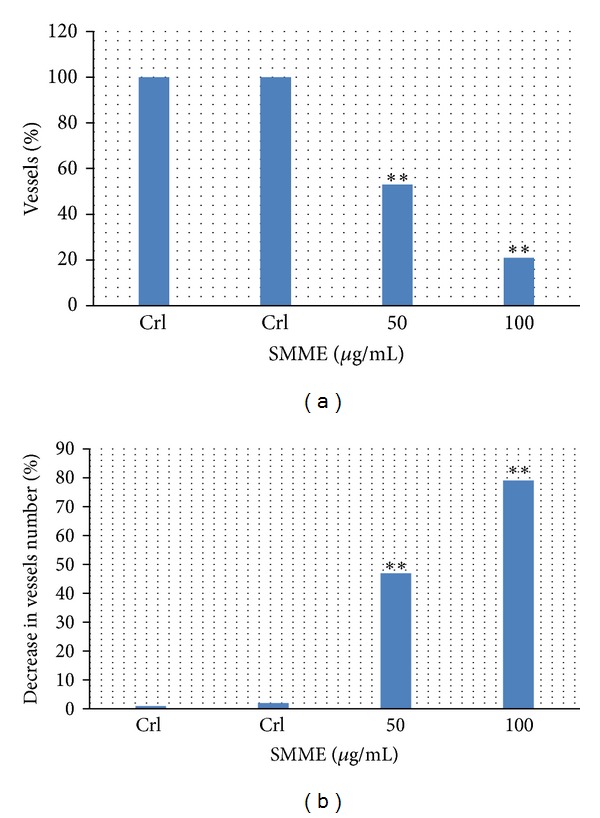
Inhibitory effect of SMME on vascularization of the 12-day CAM. **Note: Significantly different at  *P* < 0.05.

Since the angiogenesis processes play an important role in cancer in this research, the effect of the SMME on angiogenesis processes in the chorioallantoic membrane (CAM) is studied (Figure 6). The results showed that the SMME significantly (*P* < 0.05) decrease the length and the number of the vessel branches in the CAM-treated groups relative to the control and the sham-exposed group (Figure 7). Dias et al. in 2005 studied the effect of *Sargassum* A (a polysaccharide derived from *Stinophillum Sargassum*) on angiogenesis process, and they showed that this polysaccharide has the antiangiogenesis and anticancer properties via modulating the activity of heparin-binding angiogenic growth factors [37]. Sugawara et al. in 2006 proved that the fucoxanthin which is derived from brown seaweed has the anti angiogenic properties via restraining tube formation of the endothelial cells of the umbilical vein and had no effect on the immigration [38]. Ganesan et al. in 2010 investigated the antiangiogenesis effect of the siphonaxanthin derived from the green seaweed, and they conclude that siphonaxanthin inhibits angiogenesis both in cell culture model systems and ex vivo and demonstrated its potential as functional compound [39]. 

The fucoidan derived from the *Undaria pinnatifida* brown seaweeds, which is a kind of sulfate polysaccharide, has antiangiogenesis properties. This polysaccharide has restraining effects on cellular proliferation and immigration and the formation of vessel network. This study showed that the fucoidan decreased the growth of the blood vessels and decreased the expression of VEGF-A angiogenesis factor [40].

 Qianming et al. in 2011 study the effects of GFP08 (a kind of polysaccharide, which is derived from *Grateloupia filicina* brown seaweed) on angiogenesis in CAM. The results showed that this polysaccharide in a dosage-dependent manner decreased the formation of the new blood vessels. This polysaccharide decreased the expression of TF protein (tissue factor) without affecting MMP-2 and MM-9 activities [41].

Despite the fact that nowadays most of the studies are conducted on the purified materials, in this study, whole extracts were used rather than isolated phenols, testing the hypothesis that the naturally occurring family of compounds present in the seaweed and used in the diet might have synergistic properties. For example, in a study that was done by Fini in 2008, it was revealed that the raw materials in comparison with purified compounds show better and stronger effects in cancer cell death, which can be an indication of a sort of synergistic among them [42]. Our results showed that the treatment with SMME significantly decreases the angiogenesis in CAM, which was observed as the decrease in the length and the number of vessels in comparison with the control samples.

This study showed that SMME could inhibit the growth of cancer cells and could induce apoptosis in human breast cancer in time- and dose-dependent manner, also significantly decrease the angiogenesis in CAM. Thus, this polyphenol-rich marine brown alga, *Sargassum muticum,* might be an abundant source of potential complementary and alternative functional food for the prevention and treatment of cancer, and it is the most suitable for further research to deal with selective antitumor active substances to human cancer especially breast cancer.
